# Phylloxera (*Daktulosphaira vitifoliae* Fitch) alters the carbohydrate metabolism in root galls to allowing the compatible interaction with grapevine (*Vitis* ssp.) roots

**DOI:** 10.1016/j.plantsci.2015.02.002

**Published:** 2015-05

**Authors:** Michaela Griesser, Nora Caroline Lawo, Sara Crespo-Martinez, Katharina Schoedl-Hummel, Krzysztof Wieczorek, Miroslawa Gorecka, Falk Liebner, Thomas Zweckmair, Nancy Stralis Pavese, David Kreil, Astrid Forneck

**Affiliations:** a Division of Viticulture and Pomology, Department of Crop Sciences, University of Natural Resources and Life Sciences, Konrad Lorenz Str. 24, 3430 Tulln, Austria; b Division of Plant Protection, Department of Crop Sciences, University of Natural Resources and Life Sciences, Konrad Lorenz Str. 24, 3430 Tulln, Austria; c Department of Botany, Warsaw University of Life Sciences (SGGW), Nowoursynowska 159, 02-787 Warsaw, Poland; d Department of Chemistry, Division of Chemistry of Renewable Resources, University of Natural Resources and Life Sciences, Konrad Lorenz Str. 24, 3430 Tulln, Austria; e Department of Biotechnology, University of Natural Resources and Life Sciences, Muthgasse 18, 1190 Vienna, Austria; f Life Sciences, University of Warwick, Coventry CV4 7AL, UK

**Keywords:** ATP, adenosin-triphosphate, BLAST, basic local alignment search tool, BLAT, BLAST-like alignment tool, BSTFA, N,O-bis(trimethylsilyl)trifluoroacetamide, CFDA, carboxyfluorescein diacetate, DFCI, Dana Farber Cancer Institute, DMAP, 4-dimethylaminopyridine, FC, fold change, GC–MS, gas chromatography–mass spectrometry, GO, gene ontology, HEPES, 4-(2-hydroxyethyl)-1-piperazineethanesulfonic acid, NAD, nicotinamide adenine dinucleotide, NADH, nicotinamide adenine dinucleotide hydroxide, OD, optical density, PTFE, polytetrafluoroethylene, TMCS, trimethylsilyl chloride, qPCR, quantitative polymerase chain reaction, VvGI7, *Vitis vinifera gene index release 7*, Plant sink, Root gall, Carbohydrate, Primary metabolism, Grapevine

## Abstract

Gall forming phylloxera may compete for nutrients with meristematic tissues and develop heterotrophic structures that act as carbon sinks. In this work, we studied the underlying starch metabolism, sink-source translocation of soluble sugars towards and within root galls. We demonstrated that nodosities store carbohydrates by starch accumulation and monitored the expression of genes involved in the starch metabolic. Thereby we proved that the nodosity is symplastically connected to the source tissues through its development and that the starch metabolism is significantly affected to synthesize and degrade starch within the gall. Genes required for starch biosynthesis and degradation are up-regulated. Among the carbohydrate transporters the expression of a glucose-6-phosphate translocater, one sucrose transporter and two SWEET proteins were increases, whereas hexose transporters, tonoplast monosaccharide transporter and Erd6-like sugar transporters were decreased. We found general evidence for plant response to osmotic stress in the nodosity as previously suggested for gall induction processes. We conclude that nodosities are heterogenous plant organs that accumulate starch to serve as temporary storage structure that is gradually withdrawn by phylloxera. Phylloxera transcriptionally reprograms gall tissues beyond primary metabolism and included downstream secondary processes, including response to osmotic stress.

## Highlights

• Sucrose is transported symplastically towards developing and growing nodosities.
• Starch is accumulated and metabolized during nodosities growth and development.
• Nodosity formation has systemic effects on non-infected root tips of phylloxerated plants.
• Gall formation reprograms processes of the secondary metabolism as demonstrated transciptionally.

## 1 Introduction

Grape phylloxera (*Daktulosphaira vitifoliae* Fitch, Homoptera: Phylloxeridae) is a global pest that feeds on roots and leaves of susceptible cultivars of *Vitis* ssp. causing tremendous damage in viticulture. Numerous grape phylloxera biotypes and strains feeding on both own-rooted cultivars and rootstocks with partial resistance have been described worldwide which provide an effective and mostly sustainable solution for phylloxera management [1–5]. The insects induce a feeding site within the meristematic zone of the root tip, where they stay attached to the root, feeding both inter- and intracellularly [6]. This feeding behaviour induces changes in the uptake and transport of water, minerals and assimilates [7]. *D. vitifoliae* root galls (nodosities) are hook-shaped structures essential for the compatible interaction of the obligatory parasitic phylloxera with its host plant grapevine. Galling aphids may cause no obvious damage but manipulate solutes translocation patterns in plants and stimulate phloem differentiation within their hosts to acquire resources produced in autotrophic plant tissues which are fundamental to their survival and fitness [8]. Grapevine is a symplastic phloem loader due to the presence of plasmodesmata connecting mesophyll cells with phloem associating cells. Phloem unloading occurs within the symplast or via efflux into the apoplast and subsequent carrier-mediated uptake by sink cells expressing membrane-localized transporters [9]. Enzymatic hydrolysis of sucrose by invertase and sucrose synthase within sink organs regulates the sink strength. Information on the causes of altering sink strength in insect induced root galls is scarce and previous research showed that nodosities are altered compared to non-infected roots by accumulation of starch [10,11], soluble proteins [11,12], amino acids [13] and volatile metabolites [14]. Insect-induced plant galls are widely reputed to act as strong carbon sinks operating as physiological sinks for nutrients and assimilates and thus having elevated sucrose levels and high osmotic pressure. Generally plant cells cope with excessive amounts of sugars by the formation of starch that accumulates in plastids as water-insoluble granules. Nodosities contain large (1–100 μm) water insoluble semi-crystalline starch granules that stain blue with iodine and most possibly serve as storage material [10]. Starch metabolism involves the concerted actions of many enzymes including soluble and granule-bound starch synthases, branching enzymes, debranching enzymes isoamylase, α-glucan phosphorylases, α-amylases and ß-amylases [15]. Genomic approaches to understand the expression of genes in plants attacked by gall-forming insects are limited, but they may provide valuable data on the physiological networks, signalling and the effects of fluctuating mineral concentrations and carbohydrate partitioning for gall induction and development [16,17].

In the current study we examined the expression of genes involved in the carbohydrate metabolism in phylloxera infected roots. Basing on previous experimental results and results published in the literature [18] we postulate that nodosities form heterotrophic plant structures that: (1) are strong sink tissues with ability to increase phloem flow and symplastic transfer of sucrose; (2) accumulate starch as carbohydrate storage to compensate excess sugar levels occurring during phylloxera feeding site initiation and gall development; and (3) facilitate starch degradation to provide glucose ingested by the insect in real time. Several complimentary approaches, including transcriptomic analyses, measurements of starch and sugars contents and histological analyses of the galls were conducted to understand the mechanisms responsible for starch accumulation and to collect new data concerning mechanisms underlying phylloxera-root interactions.

## 2 Materials and methods

### 2.1 Permanent phylloxera single founder lineage

Leaf-galling phylloxera [*Daktulosphaira vitifoliae* Fitch (Hemiptera: Phylloxeridae)] were collected in Grosshoeflein (Austria) in 2007. A single founder lineage was established in the greenhouse and reared on leaf-forming rootstocks [Teleki 5C (*Vitis berlandieri* Planch. × *V. riparia* Michx.)] where they reproduced asexually. The homogeneity of population was confirmed by genotyping as previously described [19]. Teleki 5C plants from the same clone and initial population were used in all experiments.

### 2.2 Collection of nodosities

Nodosities were collected from plants cultivated in a climate chamber (16 h photoperiod at 64 μmol m^−2^ s^−1^, 24 °C and 45–55% relative humidity) in August and October 2009 as described elsewere [3]. In brief: one-node cuttings from the T5C rootstock were dipped in 0.3% indole-3-butyric acid (Seradix B2, Kwizda GmbH, Vienna, Austria) to promote rooting and placed into “Jiffy-7” pots (40 mm; Jiffy Products International AS, Norway). After six weeks, rooted plants were transferred into a 1:2 perlite:seramis mixture (SERAMIS Tonne-Granulat, Austria). Plants were inoculated with 60 sibling phylloxera eggs once several roots became visible through the plastic bottles. Nodosities were collected at noon and divided into four batches according to the developmental stage (L2, L3, L4 and L5) of the feeding aphids. Control samples consisted of uninfected root tips (length ca. 1.5 cm) of phylloxera inoculated and non-inoculated plants. The development of phylloxera larvae is strictly correlated with time after inoculation and thus nodosities with L2 correspond to 5–7 days after inoculation (dai), L3 to- 8-13 dai and, L4 to- 14-20 dai and L5 are older than 21 dai. Phylloxera larvae were carefully removed from collected samples to avoid contamination of samples with insect DNA and RNA, and the samples were immediately frozen in liquid nitrogen and stored at −80 °C.

### 2.3 Starch analysis

Accumulation and degradation rates of starch in nodosities were determined using a modified protocol [20]. Batches of nodosities associated with L2 and L5 larvae and the control uninfected root tips from phylloxera-infested plants were collected 2, 8 and 14 h into the 16 h light period to survey circadian rhythms of storage starch deposition in roots. Frozen samples (weight 0.15–0.30 g) were homogenized with a ball mill (MM400, Retsch, Haan, Germany) at 30 Hz for 30 s. Plant material was washed 3 times in boiling 80% ethanol for 5 min and centrifuged (5000 × *g* for 5 min; Heraeus Megafuge 16 R, Thermo Scientific, Langenselbold, Germany). After each wash the supernatant was discarded while the remaining pellets were resuspended. The remaining pellets were homogenized with water to reach a homogeneous weight concentration (1 g mL^−1^). The samples were heated to 100 °C for 10 min (Eppendorf Thermomixer Comfort, Germany) to gelatinize the starch granules. Each probe was split into four equal replicates and sodium acetate (pH 5.5) was added to each tube to reach a final concentration of 100 mM. Two replicates containing starch were digested to glucose by a mixture of α-amyloglucosidase (6 U; Sigma–Aldrich Ltd, Dorset, England) and α-amylase (0.5 U; Sigma–Aldrich Ltd, Dorset, England) whereas as technical control, the two other remaining probes were non-treated with enzymes. All tubes were incubated at 37 °C for 4 h and centrifuged for 10 min at 10,000 × *g* afterwards. The enzymatic glucose oxidation assay was conducted on supernatants. 50 μL of the supernatant was mixed with 150 μL master mix (buffer solution containing 100 mM HEPES, 1 mM NAD, 0.5 mM ATP, 4 mM MgCl_2_ and water), 1 U of hexokinase (Sigma–Aldrich Ltd, Dorset, England) and 1 unit of glucose 6-phosphate dehydrogenase (Sigma–Aldrich Ltd, Dorset, England). The OD of the NADH produced in the glucose oxidation assay was measured with a microplate reader (FLUOstar Omega, BMG Labtech, Ortenberg, Germany) at 340 nm. The kinetic reaction was measured at 20 repeats and starch content was calculated according to [20].

### 2.4 Carbohydrate analysis

Samples were collected according to the developmental stage of attached larvae (L2, L3, L4, L5, control root tips from uninfested and phylloxera-infested plants). Two to five biological replicates per developmental stage were collected and used for analysis after larvae removal, freezing in liquid nitrogen and storage at −80 °C. Samples were ground in a ball mill (MM400, Retsch, Haan, Germany) at 30 Hz for 30 s and lyophilized. Approximately 10 mg of freeze-dried material was used for subsequent derivatization assays.

Derivatization and analysis was carried as described by [21]. Respective samples consisting of approximately 10 mg of freeze-dried material were dissolved for 1 h in 200 μL pyridine containing methyl β-d-galactopyranoside (IS; 1 mg mL^−1^) and *O*-ethylhydroxylamine hydrochloride (40 mg mL^−1^). Afterwards, for silylation, 200 μL DMAP (1.5 mg mL^−1^) in pyridine (1.5 mg mL^−1^) and 200 μL of BSTFA containing 10% (v/v) TMCS were added. After vortexing, the sample was kept for 2 h at 70 °C and then it was allowed to cool down to room temperature. The sample was diluted with 600 μL of ethyl acetate, vortexed carefully and filtered through a PTFE membrane (pore size 0.45 μm, *Ø* = 13 mm).

GC–MS analysis was an Agilent 7890 gas chromatograph (Agilent Technologies, Santa Clara, CA, USA) equipped with a CTC-PALxt autosampler controlled by Chronos software v.3.5 (Axel Semrau, Spockhövel, Germany) and coupled with an Agilent 5975 triple axis mass selective detector (MSD) 0.2 μL aliquots of the derivatized samples were injected into the multimode-inlet using a CTC-PALxt autosampler which was controlled by Chronos software v.3.5 (Axel Semrau, Spockhövel, Germany). For qualitative analysis the obtained chromatograms were deconvoluted using AMDIS v.2.66 software. Peak assignment was performed with authentic reference compounds. Five-point internal standard calibration based on peak area was accomplished with MSD Chemstation E.02.02.1431 (Agilent Technologies, USA) and used for quantitative analysis.

### 2.5 CFDA analysis

CFDA-loading experiments were performed according to protocol of [22,23]. T5C plantlets were cultivated in 0.5 strength [24] medium supplemented with 15 g mL^−1^ sucrose. After four weeks of culture the plantlets were transferred into Petri dishes (*Ø* = 145 mm) with MS medium (250 mg L^−1^; Duchefa, M0255) supplemented with 5 g mL^−1^ sucrose poured up to ¾ of the dish surface [25]. Plants were grown at 24 °C and a 16/8 h light/dark photoperiod for the next 5–6 weeks. Then, plants with at least four root tips located on the medium-free surface of the Petri dish were infested with 30 phylloxera eggs. The infection progress was investigated daily under the stereo microscope. After successful infection (nodosities and/or leaf galls) two leaves were dissected and 1–2 μL of 5-carboxyfluorescein diacetate (75 mg mL^−1^; CFDA; Molecular Probes, Eugene, OR) was immediately applied on the wound surface. CFDA was prepared from the stock solution (1.3 g mL^−1^ in acetone) that was diluted 1:20 in dH_2_O. At least 30 biological replicates of CFDA treated leaves with respective controls (plants infested with phylloxera without CFDA application and non-infested plants treated with CFDA) were examined. After CFDA application the plants were kept in darkness in a growth chamber at 24 °C till the next day when they were examined under a Zeiss Axiovert 200 M inverted microscope (Zeiss, Hallerbergmoos, Germany) equipped with a filter set dedicated to FITC fluorescence.

### 2.6 Microscopy

Histological analysis was performed on sections of L2 nodosities (4–5 dai) collected from plants treated with CFDA as described in Section 2.5. As control nodosities from same-aged plants non-treated with CFDA were collected. The probes were sampled and subsequently sectioned in a room with dimmed light to avoid a possible loss of fluorescence. Control and CFDA-treated nodosities were dissected, immediately embedded in 5% (w/v) low melting agarose (Sigma–Aldrich, St. Louis, MO, USA) in PBS buffer and sectioned (30 μm) using a Leica VT 1000 vibratome (Leica, Wetzlar, Germany). Sections were stained with Toluidine-blue [10] and examined under a Zeiss Axiovert 200 M inverted microscope (Zeiss, Hallerbergmoos, Germany) equipped with an integrated Zeiss AxioCam MRc5 camera (Zeiss) or under an Olympus FV1000 confocal laser scanning microscope (Olympus, Tokyo, Japan).

Samples used for light and transmission electron microscopy (TEM) were fixed in a mixture of 2% (w/v) paraformaldehyde (Sigma–Aldrich, St. Louis, MO, USA) and 2% (v/v) glutaraldehyde (Fluka, Buchs, Switzerland) in 0.1 M sodium cacodylate buffer (pH 7.2) for 2 h at room temperature. Samples were washed 3 times in 0.1 M sodium cacodylate buffer, post-fixed with 2% (w/v) osmium tetroxide (Roth GmbH, Karlsruhe, Germany), and gradually dehydrated in graded ascending series of ethanol solutions for 20 min each at room temperature. The ethanol was substituted with propylene oxide (Sigma–Aldrich, St. Louis, MO, USA) and the samples were infiltrated in a graded ascending series of Spurr's resin dissolved in propylene oxide [26]. When infiltration was completed the samples were embedded in flat embedding moulds and the resin was polymerized at 70 °C for 18 h. Ultra-thin (60–90 nm thick) sections were taken with a Leica UCT ultramicrotome (Leica, Wetzlar, Germany) and collected on formvar-coated copper single slot grids. They were stained with uranyl acetate and lead citrate [26] and examined in FEI M268D ‘Morgagni’ transmission electron microscopy (FEI Corp., Hillsboro, OR, USA) equipped with an SIS ‘Morada’ digital camera (Olympus-SIS, Muenster, Germany). The images were captured using an SIS iTEM software (Olympus-SIS, Muenster, Germany) and adjusted for similar brightness and contrast, and resized using Adobe Photoshop software.

### 2.7 Microarray chip design and data analysis

An Agilent SurePrint Custom GE 4 × 44 microarray (Agilent #G2514F-031062) was designed using a comprehensive set of functionally annotated cDNAs derived from ESTs and other sources of expressed mRNAs as available through the latest release of the TIGR Gene Index hosted at the Dana Farber Cancer Institute (VVGI v7). A subset of 39.938 targets from the VVGI with either a consensus sequence or functional annotation by GeneOntology terms was selected and submitted to our probe design pipeline [27]. Total RNA was isolated from L2 and L4/L5 nodosities (9 pieces each per sample) and 18 uninfested root tips per sample using a modified RNeasy Plant Mini Kit with on column DNA digestion (Qiagen, Hilden, Germany) as described in [28]. The integrity of RNA was checked using an Agilent 2100 Bioanalyzer (Agilent, Santa Clara, CA, USA) and a Nanodrop ND-1000 spectrophotometer (NanoDrop, Wilmington, Germany). Microarray analyses were performed in a dye swap configuration using the Agilent SurePrint Custom GE 4 × 44 microarrays (Agilent #G2514F-031062) as described in detail [29]. The eight microarrays were scanned with an Agilent G2505C yielding 20-bit TIFF images. The scanned images were analyzed with Feature Extraction Software version 10.10.1.1 (Agilent) using default parameters (protocol GE2_1010_sep10). The resulting tables were read into the R statistical analysis environment (www.r-project.org) and analyzed using the *limma* package of the Bioconductor suite (www.bioconductor.org). Following internal and published studies of pre-processing options for Agilent microarrays [30], the raw median pixel intensities were used in preference for further normalization and analysis, with no background subtraction and no other preprocessing by Agilent.

As an examination of pairwise quantile–quantile plots showed only random fluctuations, inter-chip normalization could be achieved using quantile normalization [31]. After normalization, robust multi-chip models were fit using the lmFit function [32] of the Bioconductor package *limma*. The result-tables also include *q*-values as indicators of significance after conservative Benjamini–Yekutieli correction for multiple testing for strong control of the False Discovery Rate [33]. For the statistical tests, individual gene variances have been moderated using an Empirical Bayes approach that draws strength from transferring variance characteristics from the set of all genes to the test for each individual gene [32]. Further technical considerations about the microarray design and the results of a GeneOntology analyses see [29].

### 2.8 Microarray: gene annotation/ID assignment

While the DFCI Grape Gene Index still holds the most comprehensive cDNA collection based on experimental evidence to date, the associated functional annotation has been updated less frequently. Therefore, information from the VitisNet platform was used to transfer putative functional gene annotations for targets differentially expressed in our analysis by mapping the target sequences to the latest VitisNet annotation (Reference file “Gene Annotation”; http://www.sdstate.edu/ps/research/vitis/pathways.cfm) [34,35]. For all the targets differentially expressed in this study (12,088), we first sought to identify potential ID mappings. Nucleotide sequences of targets were obtained from the DFCI Grape Gene Index Release 7.0. Using the BLAT search function of the Grape Genome Database at Genoscope (http://www.genoscope.cns.fr/externe/GenomeBrowser/Vitis/), the target sequences were assigned to gene IDs in the database when they met the following criteria: sequence identity higher than 97% across the match, a single high-score hit and a sequence overlap between query and best hit of at least 75%. For selected genes of the carbohydrate metabolism and for the targets most strongly regulated, these assignments were further verified using the mutual best hit criterion by performing a reverse BLASTn search, of the obtained hit gene sequence at Genoscope versus the targets of the CFDI Grape Gene Index (*E* values < 1e^−100^), as well as by performing a BLASTp search of the gene amino acid sequence obtained at Genoscope versus the TAIR *Arabidopsis* database (www.arabidopsis.org, *E* value typically < 1e^−50^, and in some cases *E* value < 1e^−25^). Based on this mapping of target IDs and the additional annotation and information obtained from VitisNet from the best hit in *Arabidopsis thaliana.* To use the MapMan (http://mapman.gabipd.org/web/guest/mapman) software for illustration, a mapping file was established according to the corresponding *Arabidopsis* gene IDs. With this criteria 7414 differentially expressed genes of our microarray analyses could be assigned to MapMan functional categories [36–38]. Our data represent a putative annotation as detailed above as a general functional re-annotation of the genome is pending and out of scope for this manuscript.

### 2.9 qPCR

Samples were sampled 1, 3 or 7 dai at mid-photoperiod. Gene expression analyses were performed in four biological replicates per treatment [39]. RNA was isolated according to RNeasy Plant Mini Kit protocol (Qiagen, Hilden, Germany), which was supplemented with an additional step for DNA digestion (RNase-Free DNase Set, Qiagen, Hilden, Germany). Each individual sample consisted of 16–24 nodosities. A subsequent reverse transcription (500 ng RNA) was performed using a QuantiTec Reverse Transcription kit (Qiagen, Hilden, Germany). qPCR reactions were performed using the Rotor-Gene cycler (Qiagen, Hilden, Germany) employing KAPA SYBR FAST qPCR Universal (Peqlab, Erlangen, Germany) as a detector agent. All PCR reactions were performed in duplicates. All primers were tested for their efficiency prior to analysis by conducting standard curves with four step template dilutions. Efficiencies were calculated using the formula: *E* = [10^(−1/slope)^] − 1. For primer sequences see Additional file 1. Cycling conditions were as follows: one cycle for 4 min at 95 °C, 35 cycles for 5 s at 95 °C, 20 s at 60 °C, 5 s at 72 °C and 10 s at 75 °C, when the fluorescence signal was measured. Dissociation curves were performed with continuous fluorescence acquisition between 70 and 95 °C. Quantitative fold changes (FC) were calculated using the 2^−ddCt^ formula [40] using the expression of two reference genes, actin (GSVIVT01026580001) and ubiquitin 1 (GSVIVT01038617001) for sample normalization.

## 3 Results

### 3.1 Starch accumulates in nodosities

Starch content varied in plants due to environmental conditions and time of the day. As heterotrophic plant organs nodosities may either be connected to the carbohydrate partitioning regime in grapevine or act as independent plant entities which import carbohydrates only oneway for storage. To assay circadian rhythm effects, we analyzed starch contents in nodosities of L2 and uninfected root tips of phylloxera infested and uninfested grapevines at 6 am, noon and 6 pm. Although starch contents declined towards the 6 pm sampling, no significant differences among all examined time points were found in any sample analyzed (Additional file 2).

**Fig. 1:**
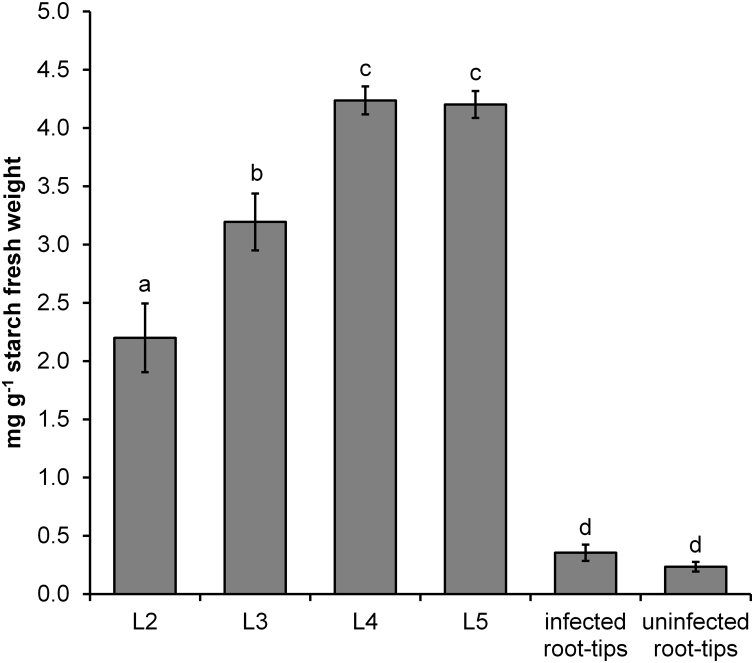
Starch content in nodosities. Starch content (mg g^−1^ fresh weight) in nodosities associated with feeding larvae at L2–L5 stage and root tips collected from phylloxera infested and uninfested plants. Data present arithmetic mean values and standard deviations from at least 3 biological replicates. Probes marked with the same letter do not differ significantly according to Tukey-HSD Test (*p* < 0.05).

The starch content in nodosities of all stages (L2, L3, L4, L5) was significantly higher than in both controls (uninfected root tips from infested and uninfested plants). Massive starch accumulation occurred early upon gall induction (L2) increased steadily with gall development (L4, L5). Starch levels in control roots were very low in both control probes and there was no significant difference between uninfected root tips collected from infested and uninfested plants. The starch content climaxed between L4 and L5, before the phylloxera started oviposition (Fig. 1).

### 3.2 Soluble sugar pools in nodosites

Since nodosities serve as nutrient pool for sedentary phylloxera, the composition of the sugar pool may be indicative of the conditions of the galls cells and the demands of the insect. Sucrose and glucose levels significantly decreased as the nodosity developed (Table 1). The uninfected root tips from phylloxera infested plants showed the highest contents of both hexoses. In general uninfected root tips from non-phylloxerated plants indicated higher amounts of glucose as compared to the nodosities, whereas the sucrose levels were much decreased in comparison to the infected plants (both galls and uninfected root tips). Myo-inositol levels have been detected in a lower range and showed no significant differences among the nodosity stages, whereas infected root tips showed the most significant amount (Table 1). Glycerol has been detected in the controls but in none of the nodosity stages. Galactose, mannose and raffinose were not detected through our analysis in any of the nodosity stages. Galactose was detected in both controls (phylloxerated: mean 2.46 μg mg^−1^; mean non infected 1.58 μg mg^−1^).

### 3.3 Symplastic sucrose transport to nodosities

**Fig. 2:**
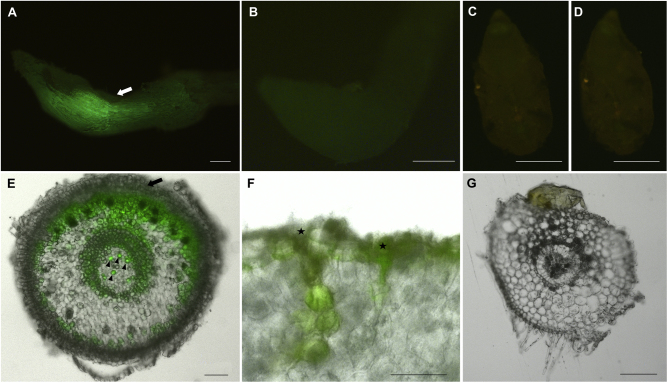
CFDA transport towards and within nodosities. In vitro plants were infected with phylloxera and the source-sink driven CFDA transport was analyzed. (A) Nodosity (4–5 dai) from CFDA-loaded plant showing CFDA fluorescence signal (green) next to incision point (arrow). (B) Nodosity (4–5 dai) from a plant non-treated with CFDA. (C) No signal was present inside phylloxera feeding on CFDA-treated root and (D) on CFDA non-treated root. (E) Section of nodosity (4–5 dai) from CFDA-treated with strong signal in phloem sieve elements (arrow heads) and in the cortical parenchyma cells that form the feeding site. Intensity of fluorescence increases gradually towards the incision point (arrow). (F) Two penetration sites (stars) with paths of CDFA-labelled cortical parenchyma cells. (G) Section of non-treated nodosity without any fluorescence, except of yellowish autofluorescence of phylloxera. Bars represent: A, B, C, D, E and G – 200 μm, F – 50 μm. (For interpretation of the references to color in this figure legend, the reader is referred to the web version of the article.)

To visualize the sink-driven transport of carbohydrates from leaves towards the nodosity and the feeding site of phylloxera a fluorescent dye carboxyfluorescein diacetate (CFDA) was used as a marker of sucrose phloem transport and symplastic phloem unloading [22,23]. According to the loading experiments CFDA was symplastically transported towards and into the nodosities associated with the L2 stage phylloxera at 4–5 dai. CFDA fluorescence signal was clearly observed in these nodosities since the first day after the dye application and it was maintained for several subsequent days (Fig. 2). The strongest fluorescence was observed next to the incision point (Fig. 2A). To exclude autofluorescence effects, control nodosities from CFDA non-treated plants were analyzed in parallel (Fig. 2B). Root tips from uninfected plants did not show any fluorescent signals in any replication (data not shown). Moreover, CFDA signals were never detected in feeding phylloxera of any larval stage as well as in control phylloxera taken from non-treated plants (Fig. 2C and D). Sections of CFDA-treated nodosities at 4–5 dai shed light on the cellular localization of the fluorescent signal within the root gall and feeding site. As expected the strongest signal was detected in phloem sieve elements from which it spread within cortex cells towards the incision point (Fig. 2E) and penetration sites (Fig. 2F). Interestingly, there was almost no CFDA fluorescence visible in the storage part of the nodosity. Non-treated cntrols of nodosities did not show any fluorescence signals beside of the faint autofluorescence (Fig. 2G).

**Fig. 3:**
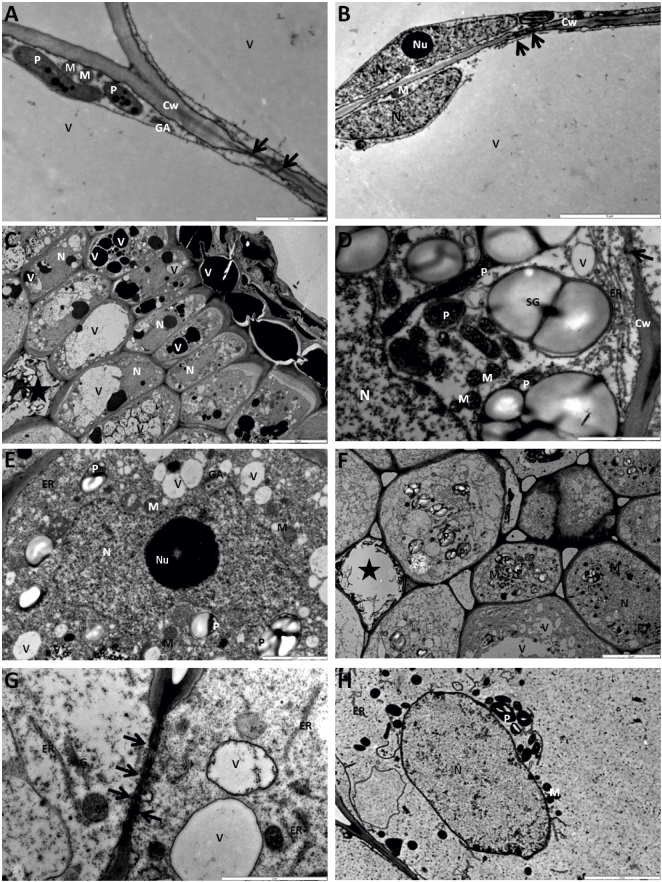
Morphology of nodosities. The ultrastructure revealed three morphological zones in nodosities of L2 and L4 phylloxera. Nodosities of L2 phylloxera are shown in photo A,B, D, H and L4 in photo C, E, F, G. (A) Part of cortex cell opposite of the feeding site with elongated plastids (P), round mitochondria and Golgi apparatus (GA) thin cell wall (Cw) with plasmodesmata (arrows) and central vacuole (V). Bar 2 μm. (B) Part of cortex cells opposite of the feeding site with thin layer of translucent cytoplasm and elongated nucleus (N) with nucleoli (Nu). Bar 5 μm. (C) Flattened in a radial direction cortex cells adjacent to the feeding site with dispersed vacuoles or vesicles. Part of them filled with electron dense materials. Star indicates perforated cells. Bar 10 μm. (D) Part of cortex cells adjacent to the feeding site with numerous elongated plastids and mitochondria. Plastids always contained starch grains (SG). The pheripheral part of the cytoplasm contained cisternal endoplasmic reticulum (ER) with ribosome attached to them. Bar 2 μm. (E) Part of cortex cells adjacent to the feeding site with enlarged and amoeboid shaped nuclei. Enlarged nucleoli were vacuolated. The plastids, mitochondria Golgi apparatus, and endoplasmic reticulum were observed in the condensed cytoplasm. Bar 2 μm. (F) Cells near the stylet sheath. The cytoplasm and nuclei became electron translucent. In the central part of the cells or around the nuclei mitochondria and plastids with starch grains were observed. Bar 10 μm. (G) The cells near the stylet sheath. Cell walls became thinner and plasmodesmata were wider (arrows). Near cell walls swollen cisternae of the endoplasmic reticulum and mitochondria appeared. Bar 2 μm. (H) The degraded cell distal to the feeding site. The cytoplasm and nuclei were electron translucent. Mitochondria and plastids with starch grains were observed. The system of inner membrane in the plastids and mitochondria seemed to be degraded. Near to nuclei swollen cisternae of the endoplasmic reticulum were observed. Bar 5 μm.

At ultrastructural level, we observed morphological cell-based alterations in nodosities relating to three particular zones of the gall (Fig. 3A–H). The zone opposite of the feeding site maintained the typical appearance of the cortex tissue with few plasmodesmata. The major altered zone, adjacent to the phylloxera feeding site showed massive morphological alterations indicating high metabolic activity (cortex cells with changed size and shape, cell wall thickened with frequently occurring plasmodesmata, strongly condensed cytoplasm with frequent starch grains). The cells comprising the zone distal to the feeding site were enlarged and degraded. Plastids contained starch grains however their stroma seemed to be degraded. The area of degraded cells was wider at L4 stage than at the stage L2.

### 3.4 Expression of genes involved in starch metabolism is affected by phylloxera feeding

The microarray analyses resulted in 6098 genes up-regulated in nodosities (log FC above 0) while 5990 genes were down-regulated (log FC below 0). The results of the GeneOntology (GO) enrichment analysis were presented in [29] and the complete results of our functional analysis are presented in Additional file 4. To assess individual biological processes, we annotated the differentially expressed genes involved in the starch metabolism pathway by identifying orthologues in *Arabidopsis* and *Vitis* during the gall induction and maturation phase of the nodosity. Among the most strongly upregulated genes (log_2_ FC > 3) we found five involved in sugar and starch metabolism: stachyose synthase, galactinol synthase, inositol-3-phosphate synthase, UDP-glucose-4-epimerase, and UDP glycosyltransferase (Table 2). In addition, we found genes involved in wound response, mainly flavonoids: chalcone isomerase, anthocyanidin 3-o-glucosyltranserase, tropinone reductase, pathogenesis related protein-4 (chitinase), leucoanthocyanidin dioxygenase (LDOX), and S-adenosyl-l-methionine:salicyclic acid carboxyl methyltransferase. Stress response genes were also affected strongly: galacturonic acid reductase, late embryogenesis abundant protein (LEA), and α-expansine 3. Of the seven most down-regulated genes (log_2_ FC < −3) the majority is involved in plant defense: two putative peroxidases, one peroxidase prx15 percursor, wound-induced protein (WI12), and pinoresinol forming dirigent protein. The biological significance of pinoresinol in plants is at present not fully understood but pinoresinol has been found to be effective as a feeding deterrent. A list of all differentially expressed genes is provided in Additional file 5.

### 3.5 Starch biosynthesis and degradation

**Fig. 4:**
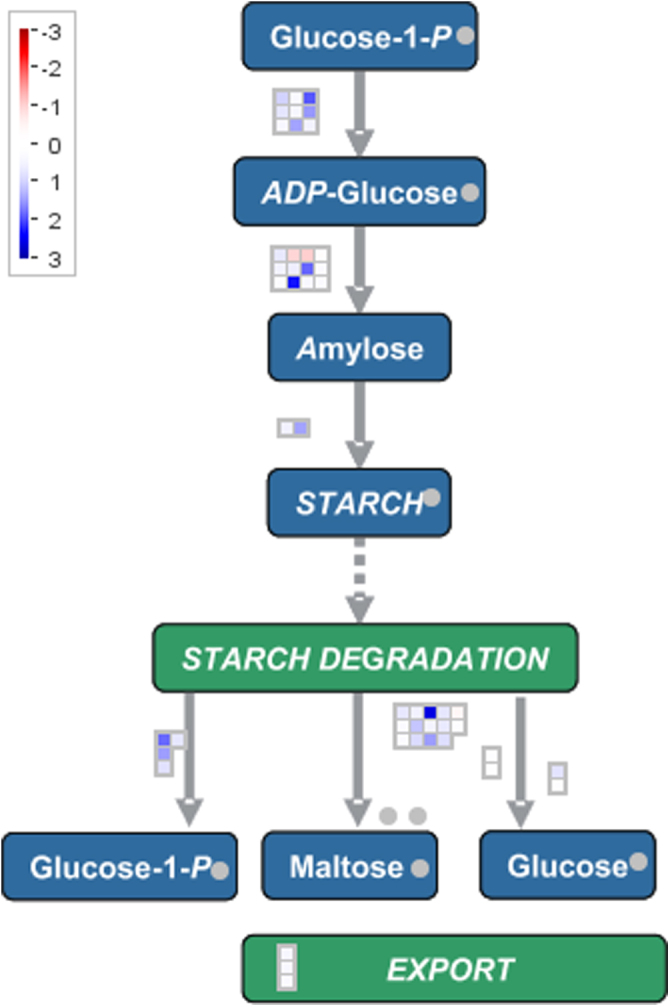
Starch metabolic pathway. Illustration of changes in expression of genes (*N* = 88) involved in the starch metabolic pathways in phylloxera–induced root galls compared to uninfected control root tips. Single squares represent one gene each (data shown in Additional file 6). The blue coloured squares indicate up-regulated genes, red boxes indicate down-regulated genes (MapMan version 3.5.1R2, [36,38]). (For interpretation of the references to color in this figure legend, the reader is referred to the web version of the article.)

Many genes involved in starch biosynthesis, such as ADP-glucose pyrophosphorylases (ADPGp), a variety of starch synthases and 1,4-α-glucan branching enzymes, were up-regulated as starch accumulated in nodosities. To confirm the microarray results, expression of several highly down- and up-regulated genes involved in starch biosynthesis and degradation was tested by qPCR. Quantitative PCR confirmed a nearly fivefold increase in the level of transcripts encoding the large subunit of ADPGp and starch synthase proteins occurring since 1 dai in nodosities and increasing with time as the nodosities develop (Table 3). Simultaneously, also expression of genes encoding proteins involved in starch degradation were increased in nodosities since 1dai in the case of ß-amylase 3 or 7 dai in the case of ß-amylase 1. ß-amylase 3 was especially strongly induced in 7dai nodosities. Starch phosphorylase was confirmed to be up-regulated in 7 dai nodosities. These results indicate that in all enzymatic steps at least one gene is up-regulated in both starch biosynthesis and starch degradation (Fig. 4). Detailed data of MapMan figures are presented in Additional file 6.

### 3.6 Sugar transport

Sugar transporter genes of six families were analyzed according to their putative functions: erd6-like (early responsive stress), ht (hexose transporters), st (sucrose transporters), vst (vacuolar tonoplast transporters), g6p (glucose-6-phosphate transporter), int (inositol transporters), pgt (plastidial glucose transporters) and sw (sucrose transporters SWEETs). The results are presented in Table 4. Among the tested hexose transporters none was up-regulated in the nodosity indicating that the monosaccharides transport does not play a major role in the carbohydrate metabolism of the gall. Instead down-regulation of expression was observed for hexose transporters VvHT8, VvHT5 and VvHT3/VvHT7, the plastidial glucose transporter VvGlcT2 and the tonoplast monosaccharide transporter VvTMT2. Additionally the expression levels of Erd6-like sugar transporters VvERD6-like5, VvERD6-like3, VvERD6-like7, VvERD6-like16, VvERD6-like8 and two inositol transporters VvINT1 and VvINT2 were down-regulated in nodosities. The sucrose transporter SUT4 was slightly upregulated in young developing nodosities 1–7 dai. Expression of SUT4 was found in all vegetative organs of the grapevine [41] although its physiological role remains open. No significant results were obtained in the microarray analyses for SUC27. Corresponding qPCR indicated an induction in nodosities at 7dai. The sucrose transporter SWEET protein family facilitates transport of neutral sugars at both the organismal and cellular level [42]. Two SWEET proteins (SWEET10 and SWEET12) studied showed significant up-regulation: SWEET10 increasing and SWEET12 decreasing with nodosity development. Expression of a putative glucose-6-phophate transporter (VvGPT2) was induced and it increased as the nodosity developed (Table 4). GPT2s belong to a group of plastidial phosphate antiporters and they occur mainly in heterotrophic tissues [43]. They mediate the import of carbon skeletons in the form of glucose-6-phosphate into plastids for starch biosynthesis (during this process inorganic phosphate is released) or as a substrate for the oxidative pentose phosphate pathway. In addition an expression of putative plastidial glucose translocator (VvGlcT2) is slightly induced in nodosities, which was shown to be involved in the export of starch degradation products.

## 4 Discussion

Aphids have evolved mechanisms to break the structural barrier of the plant cell wall during plant tissue probing and penetration. Subsequent gall induction requires dramatic changes in the plant gene expression and cellular morphology as a response to the aphid's effectors. Morphological re-arrangements and physiological changes go along with available resource. Here, evidence is presented that sugars are symplastically transported towards the nodosity and active intercellular trafficking of sugars related to the starch metabolism occur. In the present work, different approaches were taken in order to pursue a consolidated line of evidence.

### 4.1 Sink activity of nodosities

Sink activity is a physical restraint that includes multiple factors and key enzymes involved in carbohydrate utilization and storage [44]. Roots are heterotrophic carbon sinks, consisting of zones of cell division, elongation and maturation. Elongating cells have high requirements for hexoses as precursors for macromolecular carbohydrates synthesis and to allow them maintenance of high osmotic pressure necessary for turgor driven cell elongation [45]. Previous studies show that nodosities may likely exhibit both metabolic and storage sink entities to accumulate sucrose for the gall [10,46]. Sink cells either import sucrose from the apoplast directly via sucrose transporters or, alternatively, sucrose can be hydrolyzed to glucose and fructose by invertases and taken up via monosaccharide transporters. Up-regulation of sugar transporters expression may indicate the importance of apoplastic transport towards cells as shown for nematode-induced galls [23,47]. Hexose transporters are needed to transport invertase-cleaved glucose and fructose. On the other hand most woody plants are thought to be symplastic phloem “loaders” due to the presence of plasmodesmata connecting mesophyll cells with phloem-associated cells [9,48]. Our results clearly show that none of the invertase genes analyzed were regulated, indicating that invertase activity is not changed upon infection and that apoplasmic unloading plays a minor role. However, at this point apoplasmic function of low affinity transporter (e.g. SWEET 10,12) need to be further analyzed and cannot be ruled out in nodosities.

In contrast, sucrose synthase converts sucrose into fructose and UDP-glucose and may be responsible to increase the sucrose gradient and increase the amount of sugar imported for metabolism, feeding the aphids and/or storage in the root gall. Sucrose synthase requires half the net energy of the sucrose metabolic pathway catalyzed by invertases [51] and other studies confirm that sucrose synthase is involved in starch and sucrose storage sinks rather than in tissues with high cell elongation where invertases play the major role [52]. Our results indicate that sucrose synthases may be involved in nodosity formation, as a slight induction of the sucrose synthase (SUS4) was observed.

Our results clearly show that sucrose phloem unloading towards the nodosity is mainly symplastic through plasmodesmata and commence with very early nodosity development. Expression of most sucrose transporters genes showed no significantly higher expression in galled tissues if compared to root tips of uninfected roots. This is in agreement with a study by [13] employing northern blot analyses. Evidence gained from presented histological studies are in contrast to studies performed on nematode induced syncytia [53] which show mainly apoplastic transport of sucrose to the nurse cells that was in later stages additionally supported by symplastic import pathway. The induction of expression of genes coding for storage proteins and starch biosynthesis may be driven by wound-induced responses of the host plant. This has been shown in woody perennials (e.g. poplar) and it is postulated that starch deposits may serve to temporarily store carbon following tissue damage in order to protect the plant from loss of metabolites during wound response and save reserves for later recovery [54]. This may likely be the case in the nodosity since once the phylloxera is eliminated from the gall it will recover and regain full functionality as a root tip.

Sugar transporters enable intra- and intercellular trafficking, ensure sugar retrieval or act as direct sugar providers in connection with membrane-bound enzymes and thus should play pivotal roles in the nodosity's metabolism. Here, most of the sugar transporters (erd, ht, pgt, int) were either not regulated or down-regulated in nodosities indicating reduced intercellular trafficking of sugars as compared to uninfected root tips. This is possibly the result of the ceased elongation of phylloxerated root tips. SUT4 and the SWEET proteins (SWEET10, SWEET12) were up-regulated in nodosities indicating their role in gall development and/or providing sugar to the insect. The expression of a putative glucose-6-phophate transporter (GPT) (VvGPT2) was up-regulated and it increased as the nodosity developed. GPTs belong to a group of plastidial phosphate antiporters and they occur mainly in heterotrophic tissues [43]. They mediate the import of carbon skeletons in the form of glucose-6-phosphate into plastids for starch biosynthesis or as a substrate for the oxidative pentose phosphate pathway. In addition an expression of putative plastidial glucose translocator (VvGlcT) is slightly induced in nodosities, which indicates its possible role in the export of starch degradation products.

### 4.2 Storage and buffer function of nodosities

Sugars are symplastically imported into the nodosity and the feeding phylloxera excretes effectors that change the root cells and structure to develop a gall. It is assumed that turgor pressure rises and osmotic pressure drops in nodosities [55], reflecting high metabolic activity, elevated sucrose levels and the formation of starch [10,13,18].

For inducing of the specific feeding structures embedded in a root gall the L1 phylloxera pierce their rostrum and inject saliva in multiple cells adjacent to the pro-vascular system. The cells forming and acting as feeding site are significantly different from other cortical cells. The changes pertain nucleus, number and shape of mitochondria, plastids, ER Golgi apparatus fit the requirements for the nutritive tissue postulated for plant galls [55] and signify high metabolic activity. In contrast to previously described giant cells induced by plant parasitic root-knot nematodes, no cell wall ingrowth, indicating transfer cells were observed in nodosity cells. According to our present knowledge nodosities are symplastically connected to the vascular system and incoming solutes may either be taken up by the feeding phylloxera or synthetized or catalyzed by the gall's metabolism. In this paper, we have confirmed previous work showing that accumulation of starch occurs along with gene expression of the starch metabolic pathway [46]. We also have shown that the contents of sugars are significantly higher in nodosities as uninfected controls, however the concentrations decline as the nodosity matures. Given the fact that phylloxera does not directly feed on the phloem [1] Reference (1) and further may not excrete solutes via siphones, the nodosity must take the function of (1) providing nutrients to the insect by keeping a strong sink activity, (2) feeding the insect during a cycle of four moults and producing up to 200 eggs per adult and (3) buffering short term sugar excesses that occur through the physiological status of the host plant. We postulate that synthesis and degradation of starch in nodosities is triggered by feeding activity of phylloxera and that further environmental clues affect this interaction. It is noteworthy that our findings suggest that sugar could be likewisely exported towards the vascular systems to support the plant's metabolism.

## 5 Conclusion

In conclusion, the presented data supports the hypothesis that sucrose is mainly transported symplastically to nodosities and temporary accumulated to serve as carbohydrate storage structure that is gradually withdrawn by phylloxera. We did not find evidence of strong apoplastic sugar transfer in the nodosity, as most hexose transporters are down-regulated and no up-regulation of invertases was found. We showed that starch contents increased by age of the phylloxera, whereas contents of glucose and sucrose decreased as nodosities matured. Moreover, we observed systemic increase of sucrose and glucose in non-infected root tips of phylloxerated plants. Finally, we showed that phylloxera transcriptionally reprograms galled tissue beyond primary metabolism. Therefore we suggest that secondary downstream processes, including plant responses to osmotic stress are involved, in gall formation.

## Authors’ contributions

MGr supervised and discussed experiments, analyzed and interpreted microarray results, performed MapMan analyses and was responsible for the annotation of DFCI VvGI7 targets and their linkage to other *Vitis* Genome Databases, NCL collected nodosities and root samples, extracted RNA and performed qPCR analyses, SCM and KSH established and performed the starch analyses, VL performed qPCR analyses, KW performed and analyzed CFDA loading of *in vitro* plants, MG performed and analyzed ultrastructure microscopy, FL and TZ determined the sugar contents in nodosities, NSP performed the labelling and hybridization of the microarray analyses, DPK designed the Vitis Agilent microarray and analyzed the data, AF designed the research and experimental set ups and supervised all the experiments. MGr and AF discussed the results and wrote the manuscript.

## Availability of supporting data

The data supporting the results of this article are available at Array Express repository under accession No E-MTAB-2571.

## Figures and Tables

**Table 1:** Contents of primary metabolites. Results obtained for sucrose, glucose, glycerol, myo-inositol and malic acid in nodosities of L2, L3 and L4/L5 aphids and non-infected root tips from infected and non-infected plants. Data shown represent arithmetic means with standard deviations. Data were analyzed with PSAW 18 (SPSS, IBM, Westlands Center, Hong Kong) and significant differences between treatments are indicated with different letters.

	L2	L3	L4 + L5	Root tips phylloxerated plants	Root tips non-phylloxerated plants	Sign (*p* ≤ 0.05)
	Mean ± STDV	Mean ± STDV	Mean ± STDV	Mean ± STDV	Mean ± STDV	
Sucrose	171.4 ± 87.8abc	137.6 ± 70.5abc	110.5 ± 48.1b	249.5 ± 66.9c	6.3 ± 6.7d	0.003
Glucose	46.4 ± 13.3a	38.7 ± 14.7ab	14.6 ± 10.3b	125.0 ± 24.7c	70.3 ± 15.1ab	0.002
Glycerol	n.d.	n.d.		8.5 ± 4.2a	1.1 ± 0.3a	0.100
Myo-inositol	9.3 ± 3.6ab	13.5 ± 2.9a	4.0 ± 3.8b	23.7 ± 3.0c	6.9 ± 5.3ab	0.002
Malic acid	19.9 ± 15.0a	9.9 ± 7.9a	n.d.	53.3 ± 5.4a	40.1 ± 1.9a	0.013

Different letters indicate significant differences between treatments n.d. not detected

**Table 2:** Strongest regulated genes (log FC between +3 and −3) in phylloxera induced nodosities. Results represent the strongest differentially expressed genes obtained by microarray analyses of non-infected root tips and pooled samples of nodosities (L2, L5 stage phylloxera). Data represent logFC of normalized raw data of four biological replicates each.

Target ID^a^	log FC^b^	Adj. *q* value^c^	Rank^d^	Max. FP^e^	Genoscope Vitis 12xV0^f^	CRIBI 12xV1^g^	Putative gene function (VitisNet)^h^	Putative best hit *Arabidopsis* (VitisNet)^h^
TC118370	5.84	1.36E−07	16	0.00000	GSVIVT01028143001	VIT_07s0005g01680	Stachyose synthase	at4g01970
TC119411^i^	5.64	4.04E−08	1	0.00000	GSVIVT01016242001	VIT_13s0019g04590	Arsenite-transporting ATPase (ArsA)	at3g10350
TC119934^i^	5.53	1.68E−07	30	0.00001	GSVIVT01009493001	VIT_18s0001g10500	ABA 8′-hydroxylase CYP707A1	at3g19270
TC110130	5.52	4.04E−08	4	0.00000	GSVIVT01028176001	VIT_07s0005g01980	Galactinol synthase (AtGolsS1)	at2g47180
TC114480^i^	5.52	4.04E−08	2	0.00000	GSVIVT01009529001	VIT_18s0001g10820	Proteasome 26S regulatory subunit (RPN11)	at5g23540
TC127833^i^	5.12	4.04E−08	3	0.00000	GSVIVT01036279001	VIT_14s0081g00030	Pathogenesis-related protein-4 (Chitinase)	at3g04720
TC113481	4.85	8.24E−08	9	0.00000	GSVIVT01011582001	VIT_01s0011g06470	Galacturonic acid reductase	at1g59960
TC108952	4.82	1.68E−07	36	0.00001	GSVIVT01009493001	VIT_18s0001g10500	ABA 8′-hydroxylase CYP707A1	at3g19270
TC106921	4.58	3.77E−07	82	0.00003	GSVIVT01022158001	VIT_07s0031g00920	Inositol-3-phosphate synthase	at2g22240
TC121704	4.46	1.14E−07	13	0.00000	GSVIVT01008169001	VIT_17s0000g04750	Putative UDP-glycosyltransferase 89B2	at1g73880
TC118267^i^	4.21	1.43E−07	19	0.00000	GSVIVT01008073001 GSVIVT01008072001	VIT_17s0000g05550	Proton-dependent oligopeptide transport (POT) family protein	at5g62680
TC131222	4.03	2.68E−07	51	0.00001	GSVIVT01031975001	VIT_03s0063g02620	Myb RAD (Transcription factor RAD)	at4g39250
TC135159	4.00	3.95E−07	93	0.00004	GSVIVT01023002001	VIT_12s0034g01140	Plastocyanin domain-containing protein	at2g02850
TC134045^i^	3.91	7.16E−08	8	0.00000	GSVIVT01032619001	VIT_13s0067g03820	Chalcone flavonone isomerase	at3g55120
TC136295	3.84	6.02E−08	5	0.00000	GSVIVT01019892001	VIT_02s0025g04720	Leucoanthocyanidin dioxgenase	at4g22880
TC105219	3.72	3.80E−07	85	0.00003	GSVIVT01013845001	VIT_16s0013g00240	Unknown protein	at5g51110
TC117069	3.69	1.54E−07	20	0.00000	GSVIVT01015859001	VIT_03s0017g02000	UDP-glucosyltransferase	at3g21760
TC107536	3.68	4.32E−07	98	0.00004	n.a.	VIT_00s0323g00060	Putative invertase/pectin methylesterase inhibitor	n.a.
TC112713	3.51	1.08E−06	285	0.00031	GSVIVT01017247001	VIT_09s0002g06070	Late embryogenesis abundant protein	at1g52690
EE065926^i^	3.39	1.42E−07	17	0.00000	GSVIVT01016487001	VIT_13s0019g02180	Tropinone reductase	at5g06060
EC941770	3.33	6.66E−07	170	0.00011	GSVIVT01019892001	VIT_02s0025g04720	Leucoanthocyanidin dioxgenase	at4g22880
TC106256	3.24	1.68E−07	22	0.00000	GSVIVT01018903001	VIT_04s0023g02290	S-adenosyl-l-methionine:salicylic acid carboxyl methyltransferase	At1g19640
TC111440	3.23	7.28E−07	182	0.00013	GSVIVT01007987001	VIT_17s0000g06360	Alpha-expansin (AtEXPA1)	at1g69530
TC114155	3.13	7.16E−08	7	0.00000	GSVIVT01019833001	VIT_02s0025g04210	UDP-glucose 4-epimerase (AtUGE1)	at1g12780
TC122320	3.07	6.66E−07	171	0.00011	GSVIVT01031769001	VIT_03s0063g00710	Alpha/beta-hydrolases superfamily protein	at1g47480
TC131023	3.02	1.42E−07	18	0.00000	GSVIVT01011638001	VIT_01s0011g05920	S-adenosyl-l-methionine:salicylic acid carboxyl methyltransferase	at1g68040
TC129545	−3.02	1.68E−07	29	0.00000	GSVIVT01007448001	VIT_00s0567g00020	Peroxidase	at1g49570
TC115365	−3.06	9.67E−07	268	0.00026	GSVIVT01030243001	VIT_08s0058g00800	Wound-induced protein WI12	at5g01740
TC123291	−3.06	1.32E−07	15	0.00000	GSVIVT01036085001	VIT_06s0080g00840	Integral membrane family protein	at2g27370
TC121390	−3.08	4.61E−07	112	0.00005	GSVIVT01009902001	VIT_18s0001g14270	Gibberellin-regulated protein 1 (GASA1)	at1g75750
TC133463	−3.20	7.14E−07	178	0.00013	GSVIVT01029241001	VIT_11s0052g00650	Peroxidase	at2g18980
TC132265	−3.31	8.80E−07	230	0.00020	GSVIVT01019452001	VIT_02s0025g00750	Pinoresinol forming dirigent protein; disease resistance response family protein	at4g23690
TC129746	−3.40	7.16E−08	6	0.00000	GSVIVT01010080001	VIT_01s0010g00390	Peroxidase prx15 precursor	at1g30870

a Gene ID obtained from DFCI Gene Index VvGI7.

b Column log FC approximates the nominal log_2_-fold change, which can be used to compare or rank effect strength within this list. See Section 2 for details. Note that without external calibration controls these values cannot be compared to log fold-change values from other experiments.

c Column Adj. *q* value gives the adjusted *q*-value. See Section 2 for details. In the full list, this number indicates a statistically conservative estimate for the False Discovery Rate in the list of candidates so far as ordered by evidence strength (rank 1 to current rank).

d Column Rank indicates ranking of candidate genes by evidence for differential expression.

e Column Max. FP combines the two previous columns to estimate the max. number of False Positives to expect in the list of candidates so far (rank 1 to current rank).

f Putative best hit by BLAT search at the Genscope Genome Database release from 19 March 2010 at (http://www.genoscope.cns.fr/externe/GenomeBrowser/Vitis/).

g Putative best hig by BLAST search at CIBRI Genome Database release from 5 April 2012 at (http://genomes.cribi.unipd.it/grape/).

h Putative gene function as previously determined and available as download from (http://www.sdstate.edu/ps/research/vitis/pathways.cfm) [35].

i Putative linkage of the DFCI VvGI7 ID to the Genoscope and CIBRI database. Mutual best hit analyses resulted in more than one hit. Additional detailed sequence similarity analyses will be necessary to ensure the validity of the proposed likely linkage between databases. n.a. not assigned in Genoscope 12x Genome coverage.

**Table 3:** Differentially regulated genes of starch metabolic pathway. Results of the microarray analyses of pooled samples of nodosities associated with L2 and L5 stages phylloxera in relation to unifested root tips from phylloxerated plants are shown, as well as qPCR results of nodosities from L2 stage phylloxera harvested one, three and seven days after inoculation (dai). Microarray data represent logFC of normalized raw data of four biological replicates each. qPCR data represent fold changes (FC; 2^−ΔΔC**t**^) of three biological replicates and were normalized using reference genes actin (GSVIVT01026580001) and ubiquitin1 (GSVIVT01038617001).

Target ID^a^	Genoscope Vitis 12xV0^d^	Putative gene function (VitisNet)^e^	Microarray (L2–L5)		qPCR (mean FC)		
			LogFC^b^	Adj. *q* value^c^	L2 (1dai)	L2 (3dai)	L2 (7dai)
TC108652	GSVIVT01013272001	1,4-α-d-glucan maltohydrolase, β-amylase3 BAM3 (at4g17090)	2.62	1.18E−06	4.6**	7.3*	26.9*
TC108817	GSVIVT01024804001	α-glucan phosphorylase, H isozyme PHS2 (at3g46970)	1.86	8.11E−07	1.1	1.6	2.4*
TC106333^f^	GSVIVT01001863001	β-amylase1 BAM1 (at3g23920)	1.15	6.58E−06	1.2**	1.2	2.7
TC106760	GSVIVT01020935001	α-1,3-glucosidase RSW3 (at5g63840)	0.30	0.0024	1.3	1.8	6.6
TC132465	GSVIVT01020069001	α-amylase isozyme C2 precursor, α-amylase 3 AMY3 (at1g69830)	0.57	8.05E−05	0.9	1.1	1.7
TC116845	GSVIVT01008714001	1,4-α-d-glucan glucanohydrolase, α-amylase 2 AMY 2 (at1g76130)	0.29	0.0195	0.9	1.3	1.6
TC116160	GSVIVT01035168001	Isoamylase isoform 1 ISA1 (at2g39930)	0.32	0.0028	1.4*	1.4	2.3**
TC121488^f^	GSVIVT01023805001	ADP-glucose pyrophosphorylase large subunit 1APL3 (at4g39210)	1.53	4.29E−05	4.3*	4.9	7.1
TC131870^f^	GSVIVT01015018001	Sucrose synthase SUS4 (at3g43190)	0.99	1.46E−05	n.d.	n.d.	n.d.
TC130851	GSVIVT01019680001	Starch synthase GBSS1 (at1g32900)	2.33	3.54E−07	3.8	4.1	5.9
TC115213	GSVIVT01011700001	Phosphoglucomutase, cytoplasmic PGM3 (at1g23190)	2.38	8.84E−07	1.9**	2.9*	6.0**
TC117934	GSVIVT01018452001	Phosphoglucomutase chloroplast precursor PGM1 (at5g51820)	0.48	0.0010	1.0	1.2	2.0
TC107518	GSVIVT01009899001	Hexokinase HXK3 (at1g47840)	0.50	0.0004	0.9*	1.0	1.1

a Gene ID obtained from DFCI Gene Index VvGI7.

b Column logFC approximates the nominal log_2_-fold change, which can be used to compare or rank effect strength within this list. See Section 2 for details. Note that without external calibration controls these values cannot be compared to log fold-change values from other experiments.

c Column Adj. *q* value gives the adjusted *q*-value. See Section 2 for details. In the full list, this number indicates a statistically conservative estimate for the False Discovery Rate in the list of candidates so far as ordered by evidence strength (rank 1 to current rank).

d Putative best hit by BLAT search at the Genscope Genome Database release from 19 March 2010 at (http://www.genoscope.cns.fr/externe/GenomeBrowser/Vitis/).

e Putative gene function as previously determined and available as download from (http://www.sdstate.edu/ps/research/vitis/pathways.cfm) [35]; putative *Arabidopsis* best hit in brackets.

f Putative linkage of the DFCI VvGI7 ID to the Genoscope and CIBRI database. Mutual best hit analyses resulted in more than one hit. Additional detailed sequence similarity analyses will be necessary to ensure the validity of the proposed likely linkage between databases.

* Indicates a statistical significant different value from FC = 1 at the level *p* ≤ 0.05.

** Indicates a statistical significant different value from FC = 1 at the level *p* ≤ 0.001. n.d. not determined.

**Table 4:** Differentially regulated sugar transporter genes. Results of the microarray analyses of pooled samples of nodosities associated with L2 and L5 stages phylloxera in relation to unifested root tips from phylloxerated plants are shown, as well as qPCR results of nodosities from L2 stage phylloxera harvested one, three and seven days after inoculation (dai). Microarray data represent log FC of normalized raw data of four biological replicates each. qPCR data represent fold changes (FC; 2^−ΔΔCt^) of three biological replicates and were normalized using reference genes actin (GSVIVT01026580001) and ubiquitin1 (GSVIVT01038617001).

Target ID^a^	Genoscope Vitis 12xV0^d^	Putative gene function (VitisNet)^e^	Microarray (L2–L5)		qPCR (mean FC)		
			Log FC^b^	Adj. *q* value^c^	L2 (1dai)	L2 (3dai)	L2 (7dai)
TC130780	GSVIVT01015361001	Plastidial glucose transporter VvGlcT2 (at1g67300)	0.17	0.0425	0.9	1.0	1.1
TC134625^f^	GSVIVT01003181001	Hexose transporter VvHT8 (at1g11260)	−1.36	7.56E−06	0.6	0.7*	0.4**
TC104920	GSVIVT01017937001	Hexose transporter VvHT5 (at5g26340)	n.s.	n.s.	0.8	0.9	2.0
TC108578	GSVIVT01001036001	Hexose transporter VvHT3/VvHT7 (at4g02050)	−0.97	1.11E−05	0.7*	0.7*	0.9
TC113866	GSVIVT01023868001	Tonoplast hexose transporter VvTMT2 (at4g35300)	−0.32	0.0084	0.7	0.7	0.7*
TC118374	GSVIVT01022034001	ERD6-like transporter Vverd6-like5 (at1g54730)	−0.36	0.0010	n.d.	n.d.	n.d.
TC112781^f^	GSVIVT01022026001	ERD6-like transporter Vverd6-like3 (at1g08920)	−0.86	4.52E−05	n.d.	n.d.	n.d.
TC110704	GSVIVT01011047001	ERD6-like transporter Vverd6-like7 (at2g48020)	−0.56	0.0001	n.d.	n.d.	n.d.
TC123985	GSVIVT01017836001	ERD6-like transporter Vverd6-like16 (at5g18840)	−1.36	3.39E−06	1.6	1.0	0.9
TC126484	GSVIVT01022022001	ERD6-like transporter Vverd6-like8 (at3g05150)	−0.77	1.23E−05	n.d.	n.d.	n.d.
TC115303	GSVIVT01010741001	Inositol transporter VvINT1 (at2g43330)	−0.65	0.0004	1.0	0.7	0.8
TC113429	GSVIVT01021530001	Inositol transporter VvINT2 (at1g30220)	−0.71	0.0113	0.9*	1.1	1.5**
TC104946	GSVIVT01034886001	Sucrose transporter VvSUC27 (at1g22710)	n.s.	n.s.	0.7**	0.9	1.8
TC104917	GSVIVT01009254001	Sucrose transporter VvSUT4/VvSUC11 (at1g09960)	0.74	0.0001	1.8	1.5	2.0
TC111673^f^	GSVIVT01012648001	Glucose-6-phosphate translocater VvGPT2 (at1g61801)	1.29	0.0004	2.9**	3.1**	3.0**
TC107241	GSVIVT01008595001	Sugar transporter SWEET10 (at5g50790)	1.37	1.33E−05	2.7	3.7	8.8**
n.a.	GSVIVT01008597001	Sugar transporter SWEET12 (at5g50790)	n.a.	n.a.	5.0	3.1	2.5

a Gene ID obtained from DFCI Gene Index VvGI7.

b Column log FC approximates the nominal log_2_-fold change, which can be used to compare or rank effect strength within this list. See Section 2 for details. Note that without external calibration controls these values cannot be compared to log fold-change values from other experiments.

c Column Adj. *q* value gives the adjusted *q*-value. See Section 2 for details. In the full list, this number indicates a statistically conservative estimate for the False Discovery Rate in the list of candidates so far as ordered by evidence strength (rank 1 to current rank).

d Putative best hit by BLAT search at the Genscope Genome Database release from 19 March 2010 at (http://www.genoscope.cns.fr/externe/GenomeBrowser/Vitis/).

e Putative gene function as previously determined and available as download from (http://www.sdstate.edu/ps/research/vitis/pathways.cfm) [35]; putative *Arabidopsis* best hit in brackets.

f Putative linkage of the DFCI VvGI7 ID to the Genoscope and CIBRI database. Mutual best hit analyses resulted in more than one hit. Additional detailed sequence similarity analyses will be necessary to ensure the validity of the proposed likely linkage between databases.

* Indicates a statistical significant different value from FC = 1 at the level *p* ≤ 0.05.

** Indicates a statistical significant different value from FC = 1 at the level *p* ≤ 0.001 n.a. not assigned, microarray target not identified. n.d. not determined. n.s. not significant differentially expressed at *p* ≤ 0.05.
