# Supplementary material for: Phylloxera (Daktulosphaira vitifoliae Fitch) alters the carbohydrate metabolism in root galls to allowing the compatible interaction with grapevine (Vitis ssp.) roots
Source: Plant Sci. 2015 May;234:38–49. doi: 10.1016/j.plantsci.2015.02.002 (PMC4388344; doi:10.1016/j.plantsci.2015.02.002)
Supplement: Supplementary file 1 [file mmc1.pdf]

Additional file 1.pdf: Table S1. Nucleotide sequences of primers used for qPCR. The two reference genes ubiquitin and actin were used for normalization. All primers with annealing temperatures of 60°C and used with 200 nM final concentration each. Accession numbers according to 12Xv0 at Genoscope Database (http://www.genoscope.cns.fr/externe/GenomeBrowser/Vitis/).

**Gene function**	**Genoscope Vitis 12xV0**	**Primer**
Ubiquitin1 (UBQ-L40)	GSVIVT01038617001	For:5'TTGATGCAATTGGCTAGGAA3'<br>Rev:5'TGTAACACTGCATGCACCAA3'
Actin	GSVIVT01026580001	For:5'TGTGCTTAGTGGTGGGTCAA3'<br>Rev:5'ATCTGCTGGAAGGTGCTGAG3'
Sucrose synthase VvSUS4 (at3g43190)	GSVIVT01015018001	For:5'TCGCGTGAATGTGAGTGCGCTT3'<br>Rev:5'AAAGAGTTGGGCGAGGGACGGA3'
Cell wall apoplastic invertase	GSVIVT01016869001	For:5'AACCCACCAGCCTTACAGAA3'<br>Rev:5'CTGACCAGCAGCCATTGATA3'
Phosphoglucomutase chloroplast precursor VvPMG1 (at5g51820)	GSVIVT01018452001	For:5'CCCACAAAGCCGATTGAAGGCCA3'<br>Rev:5'CGGCCATCACCGCCTAAAACCA3'
Phosphoglucomutase, cytoplasmic VvPMG3 (at1g23190)	GSVIVT01011700001	For:5'CCGCCTCTCGGGAACTGGCT3'<br>Rev:5'CGGCCATCACCGCCTAAAACCA3'
Starch synthase VvStSyn (at1g32900)	GSVIVT01019680001	For:5'GCATGTCAACGGCACCCAGGAA3'<br>Rev:5'CCTCCCCACAGTTGTCCTCGC3'
ADP-glucose pyrophosphorylase large subunit 1 VvAPL3 (at4g39210)	GSVIVT01023805001	For:5'GCCTGGGGTTGCTTGCTCTGT3'<br>Rev:5'CCTCCAACTGGAACAGCAGGCG3'
Hexokinase VvHXK3 (at1g47840)	GSVIVT01009899001	For:5'CTTTGGGAAGAGAACCGTGGTGGC3'<br>Rev:5'GGAGTTTGAAGCAGCCAAGAGAGCA3'
UTP-glucose-1-phosphate uridylyltransferase, UDP-Glu-pyrophosphatase	GSVIVT01026563001<br>GSVIVT01026562001<br>GSVIVT01026561001	For:5'GGCTTGTGGAGGCTGATGCACT 3'<br>Rev:5'AGGAAGAAAACGGGAGCGGGGA3'
1,4-α-D-glucan maltohydrolase, β-amylase3, VvBAMY3 (at4g17090)	GSVIVT01013272001	For:5'TGGGCCCCTGTGGAGAGTTGAG3'<br>Rev:5'CCCACTGCGTCTGCTGATGCTT3'
α-Glucan phosphorylase, H isozyme, starch	GSVIVT01024804001	For:5'GGTGCCACAGCAGATGAAGTCCC3'

phosphorylase VvPHS2 (at3g46970)		Rev:5'AGCCAGGGAAGTCGTGACCAACA3'
B-Amylase 1 VvBMY1 (at3g23920)	GSVIVT01001863001	For:5'GGACCAGCAGGTGAGTTCCGC 3'<br>Rev:5'GGGACCGGTGCTACCCCATTCT3'
α-1,3-Glucosidase RSW3 (at5g63840)	GSVIVT01020935001	For:5'GTGGGCGGACGATTCGGCAT3'<br>Rev:5'ACATCACCGTCACGCTCCCG3'
α-Amylase isozyme C2 precursor, α-amylase 3 VvAMY3 (at1g69830)	GSVIVT01020069001	For:5'AAGCCTCCAGGGGTTGTTGGCT3'<br>Rev:5'AAGAAAACTGCTGGCGTCCCAGGAT3'
1,4-α-D-Glucan glucanohydrolase, α-amylase 2 VvAMY2 (at1g76130)	GSVIVT01008714001	For:5'GGAAGGAGCAGGACATTCACAGTCG3'<br>Rev:5'GGGTCCACTCCCTGCCTCCTG'
Isoamylase isoform 1 ISA1 (at2g39930)	GSVIVT01035168001	For:5'TGGCATGTTTCCTCACTGGGGTCT3'<br>Rev:5'AGGGCTCCCACAAAGGCATTCAG3'
Hexose transporter VvGLcT2 (at1g67300)	GSVIVT01015361001	For:5'TCGGACCTCGCAAATGTGTT 3'<br>Rev:5'AAAGAACTCGCACCAGCTACT3'
Hexose transporter VvHT3/VvHT7 (at4g02050)	GSVIVT01001036001	For:5'GCGGGCCGAAGAAGACCACT3'<br>Rev:5'CGACCCGAAAGAAGCATCGCCA3'
Hexose transporter VvHT5 (at5g26340)	GSVIVT01017937001	For:5'GGAGACCCGATCAGCAGGGCA3'<br>Rev:5'ACATGATGAAGACCCAGCCGGAGA3'
Hexose transporter VvHT8 (at1g11260)	GSVIVT01003181001	For:5'GAATTTCTGGTGGGGTCACGTCCAT3'<br>Rev:5'AGGCCACCAGCGACGAGAGA3'
Tonoplast hexose transporter VvTMT2 (at4g35300)	GSVIVT01023868001	For: 5'TCTTTCCCACCCGTGTCCGAGG3'<br>Rev: 5'GCCAAAGACACCAGCAAGGCCA3'
Sucrose transporter VvSUC27 (at1g22710)	GSVIVT01034886001	For:5'GCTGCCGGCTCCTTCAGCAA3'<br>Rev:5'TGCACGGTGGCGAATGCGAT3'
Sucrose transporter VvSUT4/VvSUC11 (at1g09960)	GSVIVT01009254001	For:5'TTTGCGGTCCCCTCTCGGGT3'<br>Rev:5'GTCGGCACCGTCACCAAGCA3'
Glucose-6-phosphate translocater VvGPT2 (at1g61801)	GSVIVT01012648002	For:5'ACCCTCTTTCCTGTTGCAGTAGCTCA3'<br>Rev:3'GACCGGCACCGGAAAGGTCTC3'
Sugar transporter SWEET10 (at5g50790)	GSVIVT01008595001	For:5'TGTTCCCTACGTGGTTGCAT3'<br>Rev:3'CCTAGCCTTCTTCGGTGCAT3'
Sugar transporter SWEET12 (at5g50790)	GSVIVT01008597001	For:5'CTGTTGGATGGAGATTGGTGATTC3'<br>Rev:3'CCAAAGACGAAACCCAGTGTG3'
Inositol transporter VvINT1 (at2g43330)	GSVIVT01008595001	For:5'GGCATACCGGGGAATTTGTG'

		Rev:3'ACAGCCACACCTGCAAGAAT'
Inositol transporter VvINT2 (at1g30220)	GSVIVT01008597001	For:5'TCAGTTGTGGCCCTGTTCTT'<br>Rev:3'GAGTCTGGCCTTTTCTCCCA'
