# Supplementary material for: Phylloxera (Daktulosphaira vitifoliae Fitch) alters the carbohydrate metabolism in root galls to allowing the compatible interaction with grapevine (Vitis ssp.) roots
Source: Plant Sci. 2015 May;234:38–49. doi: 10.1016/j.plantsci.2015.02.002 (PMC4388344; doi:10.1016/j.plantsci.2015.02.002)
Supplement: Supplementary file 2 [file mmc2.pdf]

Additional file 2.pdf: Table S2. Diurnal regulation of starch content in L2 nodosities.

The starch content in L2 nodosities and phylloxerated root tips harvested at three sampling times (6am, noon, 6pm) was determined. Three biological replicates each were analyzed (n=3). Data shown represent arithmetic means and standard deviations. Samples followed by the same letter are not significantly different according to Tukey-HSD Test ($p \leq 0.05$).

**Sample**	**Starch content (mg g^{-1} fresh weight) mean**	**Standard Deviation**
L2 nodosities (6 am)	2.167[a]	0.374
L2 nodosities (noon)	2.199[a]	0.589
L2 nodosities (6 pm)	1.349[a]	0.075
Phylloxerated root tips (6 am)	0.099[b]	0.029
Phylloxerated root tips (noon)	0.235[b]	0.082
Phylloxerated root tips (6 pm)	0.154[b]	0.046
