# Supplementary material for: Phylloxera (Daktulosphaira vitifoliae Fitch) alters the carbohydrate metabolism in root galls to allowing the compatible interaction with grapevine (Vitis ssp.) roots
Source: Plant Sci. 2015 May;234:38–49. doi: 10.1016/j.plantsci.2015.02.002 (PMC4388344; doi:10.1016/j.plantsci.2015.02.002)
Supplement: Supplementary file 3 [file mmc3.pdf]

Additional file 3.pdf: Table S3. Low molecular weight metabolites. Table of 148 low molecular weight metabolites including sugars, sugar metabolites and amino acids analyzed by GC-MS.

No.	Compound
1	Sedoheptulose
2	Methylglyoxal
3	Glycerinaldehyde
4	Threose
5	Erythrose
6	Raffinose
7	Apiose
8	Arabinose
9	2-Deoxy-D-Glucose
10	Tagatose
11	Fructose
12	Talose
13	Xylobiose
14	Lactulose
15	Trehalose
16	2-Deoxy-D-Ribose
17	Lyxose
18	Fucose
19	Allose
20	Gulose
21	Galactose
22	Sucrose
23	Cellobiose
24	Palatinose
25	Digitoxose
26	Xylose
27	Ribose
28	Sorbose
29	Mannose
30	Glucoheptose
31	Lactose
32	Turanose
33	Melibiose
34	Glycoaldehyde
35	Dihydroxyacetone
36	Rhamnose
37	Altrose
38	Maltulose
39	Gentiobiose
40	Erythrulose
41	Ribulose
42	Psicose
43	Glucose

44	Leucrose
45	Maltose
46	Glycerol
47	Threitol
48	meso-Erythritol
49	Xylitol
50	Arabitol
51	Ribitol
52	Pinitol
53	Mannitol
54	Sorbitol
55	Dulcitol
56	myo-Inositol
57	Lactitol
58	Cellobiitol
59	Maltitol
60	Galactinol
61	Glyoxylic acid
62	Pyruvic acid
63	Levulinic acid
64	2-keto-gulonic acid
65	Fructose-6-phosphate
66	Glucose-6-phosphate
67	Alanine
68	Valine
69	Norvaline
70	Leucine
71	Proline
72	Isoleucine
73	Glycine
74	Serine
75	Threonine
76	Methionine
77	Aspartic acid
78	Glutamic acid
79	Phenylalanine
80	Asparagine
81	Lysine
82	Tryptophane
83	Caproic acid
84	Oxalic acid
85	Heptanoic acid
86	Malonic acid
87	Octanoic acid
88	Succinic acid
89	Fumaric acid
90	Nonanoic acid
91	Glutaric acid
92	Decanoic acid
93	Mandelic acid
94	Malic acid

95	Salicylic acid
96	trans-Cinnamic acid
97	Pimelic acid
98	Tartaric acid
99	Suberic acid
100	Gentisic acid
101	Azelaic acid
102	Citric acid
103	Sebacic acid
104	Acetosyringone
105	Pentadecanoic acid
106	Ascorbic acid
107	Palmitic acid
108	Sinapic acid
109	Butyric acid
110	Lactic acid
111	Sorbic acid
112	Benzoic acid
113	Maleic acid
114	Citraconic acid
115	Lactic acid dimer
116	Mesaconic acid
117	Hydrocinnamic acid
118	Adipic acid
119	Ribonic acid
120	Vanillic acid
121	Terephthalic acid
122	Myristic acid
123	Quinic acid
124	Syringic acid
125	p-Coumaric acid
126	Gallic acid
127	Glucuronic acid
128	Ferulic acid
129	Caffeic acid
130	Stearic acid
131	Chlorogenic acid
132	Glycolic acid
133	Glyceric acid
134	Tartronic acid
135	Threonic acid
136	Xylonic acid
137	Gulonic acid
138	Gluconic acid
139	Galacturonic acid
